# Solitary pancreatic metastasis of gastric cancer with synchronous pancreatic ductal carcinoma: A case report

**DOI:** 10.1016/j.ijscr.2020.04.006

**Published:** 2020-05-07

**Authors:** Yuichiro Yokoyama, Hiroki Sakata, Toshimasa Uekusa, Yusuke Tajima, Masahiro Ishimaru

**Affiliations:** a Department of Surgery, Douai Memorial Hospital, 2-1-11 Yokoami, Sumida-ku, Tokyo, 130-8587, Japan; b Department of Surgery, Kanto Rosai Hospital, 1-1 Kizukisumiyoshicho, Nakahara-ku, Kanagawa, 211-8510, Japan; c Department of Pathology, Kanto Rosai Hospital, 1-1 Kizukisumiyoshicho, Nakahara-ku, Kanagawa, 211-8510, Japan

**Keywords:** CT, computed tomography, Pancreatic metastasis, Gastric cancer, Pancreatic cancer

## Abstract

**Background:**

There have been a few reports on solitary pancreatic metastases of gastric cancer. We present a case of solitary pancreatic metastasis of gastric cancer with synchronous primary pancreatic ductal carcinoma.

**Presentation of Case:**

An 86-year-old man who had undergone total gastrectomy two and half years prior presented with a poorly enhanced tumor in the pancreatic body. We diagnosed pancreatic ductal carcinoma and performed distal pancreatosplenectomy. Histopathologically, the tumor comprised poorly differentiated adenocarcinoma cells mixed with moderately differentiated tubular adenocarcinoma cells that were compatible with metastasis of gastric cancer. There was also invasive ductal carcinoma of the pancreas. The postoperative course was uneventful. Six months after surgery, computed tomography revealed peritoneal dissemination, and he died of recurrence 10 months after surgery.

**Conclusion:**

Pancreatic metastasis of gastric cancer with synchronous primary pancreatic cancer can occur and should be considered in the differential diagnosis.

## Highlights

• Solitary pancreatic metastasis of gastric cancer is rare.
• Favourable survival after surgical resection of solitary pancreatic metastasis.
• Low frequency of pancreatic duct dilation in solitary pancreatic metastasis.
• Pancreatic metastasis of gastric cancer with primary pancreatic cancer can occur.

## 1 Introduction

Solitary pancreatic metastases of gastric cancer are extremely rare, and only a few cases have been reported [[1], [2], [3], [4], [5], [6], [7], [8], [9], [10]]. The radiological features of solitary pancreatic metastases of gastric cancer resemble those of ductal carcinoma of pancreas, rendering preoperative diagnosis difficult [11]. The clinicopathological features of pancreatic metastasis of gastric cancer have not been fully defined. Therefore, we present a case of solitary pancreatic metastasis of gastric cancer with synchronous primary pancreatic ductal carcinoma. We review previous cases and discuss the clinicopathological features of pancreatic metastasis of gastric cancer. The case report described herein is in line with the SCARE criteria [12].

## 2 Presentation of case

An 86-year old man presented with a poorly enhanced tumor of pancreatic body and dilation of the main pancreatic duct seen on computed tomography (CT). Two and half years prior, he had undergone total gastrectomy, with Roux-en-Y reconstruction for gastric adenocarcinoma in our hospital. He had no remarkable medical or family history. The primary gastric cancer was located in the cardia, measuring 9 cm in the largest diameter. Microscopically, it was a non-solid type poorly differentiated adenocarcinoma, mixed with signet-ring cell adenocarcinoma and moderately differentiated tubular adenocarcinoma invading through the submucosa. No lymphatic vessel invasion was detected; however, there was venous invasion. No lymph node metastasis was found. The gastric carcinoma was completely resected. No recurrence was detected during regular follow-up.

**Fig. 1:**
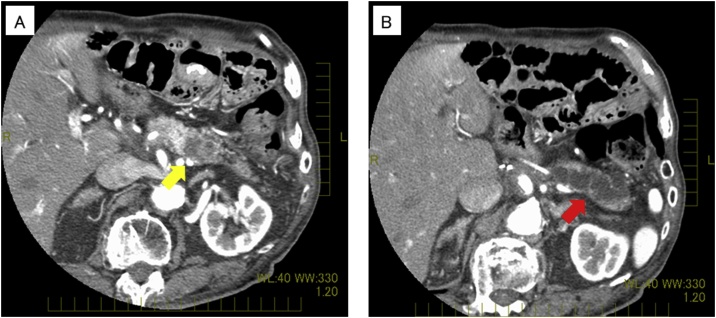
Preoperative computed tomography (CT). (a) Yellow arrow indicates poorly enhanced tumor of the pancreatic body. (b) Red arrow indicates the dilated main pancreatic duct.

**Fig. 2:**
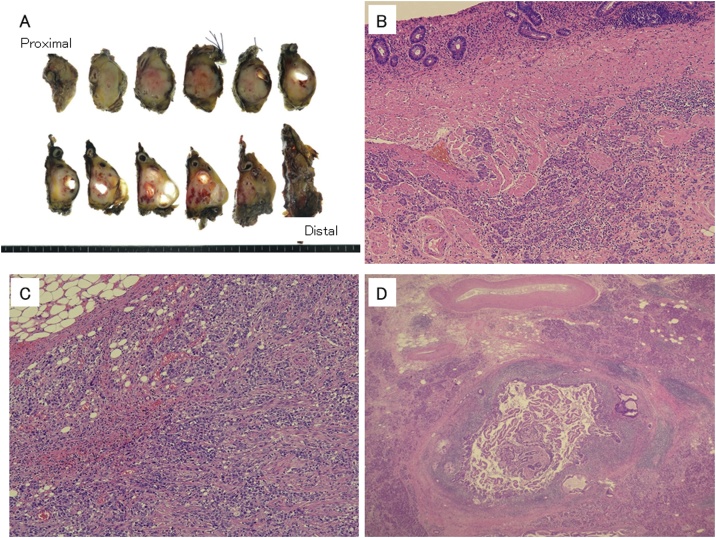
(a) Gross appearance of the resected specimen. The dimensions were 25 × 20 × 25 mm^3^ (b, c) Histopathological appearance of the primary gastric cancer (b) and resected pancreatic tumor (c). Photograph revealing that both tumors were composed of poorly differentiated adenocarcinoma cells, mixed with moderately differentiated tubular adenocarcinoma cells. (d) Histopathological appearance of invasive ductal carcinoma. Well-differentiated adenocarcinoma found in the pancreatic duct.

Two and a half years after resection, CT revealed a hypovascular mass accompanied with dilation of the peripheral pancreatic duct in the pancreatic body (Fig. 1). No other masses were detected. Because of the diagnosis of pancreatic cancer, open distal pancreatosplenectomy was performed. Intraoperative findings revealed a hard mass in the pancreatic body. The transverse mesocolon firmly adhered to the pancreas and the retroperitoneum around the pancreas had become very hard. It was very difficult to separate the pancreas from the mesocolon completely; therefore, the transverse mesocolon was partly resected together. No apparent peritoneal dissemination was detected. The resected tumor measured 25 × 20 × 25 mm^3^ and was a white to yellowish, firm mass (Fig. 2a) Microscopy revealed poorly differentiated adenocarcinoma cells mixed with moderately differentiated tubular adenocarcinoma cells whose features were inconsistent with the gastric cancer specimen resected earlier (Fig. 2b, c). The tumor cells infiltrated from this tumor into the fat tissue of the retroperitoneum around the pancreas extensively, and the surgical margin was positive because of these fat tissues. Retrospectively, this cancer cell invasion was considered the probable reason for retroperitoneum being so hard intraoperatively. No lymphatic vessel invasion was detected; however, there was venous vessel invasion. Proximal to the metastasis of the gastric cancer, 7 mm distal from the stump, well-differentiated tubular adenocarcinoma existed in the pancreatic duct and invaded surrounding tissues (Fig. 2d). This well-differentiated adenocarcinoma was near but separated from the metastasis of gastric cancer. Furthermore, the degree of differentiation was different from the gastric cancer resected before. Based on these characteristics, this well-differentiated adenocarcinoma was considered to be pancreatic ductal carcinoma. The postoperative course was uneventful. However, 6 months after surgery, CT revealed peritoneal dissemination. He died from recurrence 10 months after the surgery.

## 3 Discussion

Secondary tumors of the pancreas are rare. In autopsy surveys with malignant neoplasms, the prevalence of pancreatic metastasis has been reported to range from 1.6–11% [13]. The most common primary sites reported were lung followed by gastrointestinal tract, especially stomach, kidney, and breast [13]. Nevertheless, in the autopsy series, almost all studies included tumors that directly invaded the pancreas as secondary pancreatic tumors; this is probably the reason why the stomach has a high percentage of the primary sites of pancreatic metastasis [13,14]. When pancreatic metastasis is limited to solitary metastatic tumor in the pancreas, pancreatic metastasis of gastric cancer is extremely rare. To our knowledge, only four cases have been reported in English literature [[1], [2], [3], [4]]. Because of the high prevalence of gastric cancer in Japan, six cases have been reported in the Japanese literature; including our case, 11 cases of solitary pancreatic metastasis of gastric cancer are reviewed and discussed below (Table 1]) [[5], [6], [7], [8], [9], [10]].

**Table 1:** Histopathological features of solitary pancreatic metastasis of gastric cancer.

		Primary gastric cancer								
Author	Age/ gender	Depth	N	ly	v	Histo-logy	Duration	Preoperative Diagnosis	Pancreatic duct dilation	Prognosis
Brannigan	67/F	MP	n.d.	n.d.	n.d.	sig	120M	n.d.	(-)	12M
Nakai	59/M	n.d.	(+)	(+)	(-)	por	synchro	PK	(-)	2M died (PE)
Wente	60/F	SS	(+)	n.d.	n.d.	por	48M	PK or meta	n.d.	12M
Roland	n.d.	n.d.	n.d.	n.d.	n.d.	n.d.	n.d.	PK	n.d.	n.d.
Kondo	72/M	SS	(-)	n.d.	n.d.	mod	7M	PK or meta	(-)	72 M died
Hashimoto	77/M	SE	(+)	(+)	(+)	ud	10M	PK or meta	(-)	14M
Teshima	68/M	SS	(+)	n.d.	n.d.	mod	synchro	Intraoperative	(-)	14M
Nakae	50/F	MP	(+)	(+)	(+)	mod	12M	meta	(-)	48M
Matsumoto	72/M	SS	(+)	(+)	(-)	por	46M	IPMC	(-)	12M
Watanabe	75/M	SS	(+)	(-)	(+)	mod	48M	PK	(-)	7M
ours	86/M	SS	(-)	(-)	(+)	por	31M	IPMC	(+)	10 M died

n.d.: not documented, ud: undifferentiated adenocarcinoma, por: poorly differentiated adenocarcinoma, synchro: synchronous metastasis, PK: primary pancreatic carcinoma, meta: metastasis, PE: pulmonary emboli, IPMC: intraductal papillary mucinous carcinoma.

When gastric cancer metastasizes to the pancreas, either the hematogenous or the lymphatic pathway is considered. Of the nine patients, primary gastric cancer accompanied with lymph node metastasis occurred in seven. Lymphatic pathway has been considered to be dominant in metastasis of gastric cancer to the pancreas because the lymphatic vessel of stomach and that of pancreas are closely connected. Nevertheless, in our case, there was no lymph node metastasis and no lymphatic invasion in the primary tumor. This suggests that the pancreatic metastasis in our case was probably hematogenous metastasis from gastric cancer.

Favorable long-term survival after surgical resection of pancreatic metastasis has been reported [11]. However, the prognosis depends on the type of primary cancer. As for pancreatic resection of gastric cancer, long-term follow-up has been not discussed in many cases such as ours. Nevertheless, in almost all cases, over 1-year survival was achieved. The morbidity and mortality rates after pancreatic resection have greatly decreased in recent years; therefore, pancreatic resection should be considered when solitary pancreatic metastasis of gastric cancer is suspected [11].

The preoperative diagnoses of pancreatic metastasis have been challenging. Of the 11 cases, the diagnosis of pancreatic metastasis was made in only one case. Radiological features of pancreatic metastasis depend on the primary tumor [15]. In cases where the primary tumor is hypovascular, such as gastric cancer, it is difficult to differentiate primary pancreatic ductal carcinoma from secondary pancreatic tumors using radiological enhancement patterns because primary ductal adenocarcinoma also appears hypovascular. On the other hand, observations of pancreatic duct are important to differentiate pancreatic metastasis from ductal adenocarcinoma [16]. Ductal carcinoma originates from the pancreatic duct; therefore, disruption and dilation of pancreatic duct emerge in the early phase. Nevertheless, pancreatic metastasis occurs in the parenchyma of the pancreas and grows expansively; therefore, it probably takes longer to involve the pancreatic duct. Indeed, excluding our case, pancreatic dilation was not observed in any of the cases. The pancreatic duct dilation was observed in our case partly because the primary ductal adenocarcinoma coexisted with gastric metastatic adenocarcinoma. The coexistence of primary and metastatic adenocarcinoma in the pancreas is extremely rare, and to our knowledge, no such case has been reported. Considering the rarity of the coexistence of primary adenocarcinoma, pancreatic metastasis should be considered when a poorly enhanced pancreatic mass with no dilation of pancreatic duct is detected in patients with history of gastric cancer.

## 4 Conclusions

Solitary pancreatic metastasis of gastric cancer is rare; nevertheless, it should be considered when pancreatic tumor with no dilation of pancreatic duct is detected in patients with history of gastric cancer. Pancreatic resection should be considered when solitary pancreatic metastasis of gastric cancer is suspected.

## Declaration of Competing Interest

The authors declare that they have no competing interests.

## Funding

There is no sources of funding for our research.

## Ethics approval

Treatments for the patient were in accordance with the ethical standards of the responsible committees on human experimentation (institution and national).

## Consent

Written informed consent was obtained from the patient and the next of kin for publication of this case report and accompanying images. A copy of the written consent is available for review by the Editor-in-Chief of this journal on request.

## Authors’ contributions

YY, HS, and YT carried out the diagnosis of the tumour. YY and HS performed the surgery of this patient. TU made the histopathological diagnosis. YY and HS wrote and revised the manuscript. All authors read and approved the final manuscript.

## Registration of research studies

Our case report is not first-in-man or animal studies. So, in accordance with guidance of research registry, we do not register our case report in http://www.researchregistry.com.

## Guarantor

Yuichiro Yokoyama.

## Provenance and peer review

Not commissioned, externally peer-reviewed.

## References

[1] Brannigan A.E., Kerin M.J., O’Keane J.C., McEntee J.P. (2000). Isolated resectable pancreatic metastasis 10 years post gastrectomy. Ir. J. Med. Sci..

[2] Nakai T., Shimomura T., Nakai H. (2004). A case of isolated pancreatic metastasis of gastric cancer presenting problematic discrimination from gastropancreatic double cancer. Hepatogastroenterology.

[3] Wente M.N., Bergmann F., Fröhlich B.E., Schirmacher P., Büchler M.W., Friess H. (2004). Pancreatic metastasis from gastric carcinoma: a case report. World J. Surg. Oncol..

[4] Roland C.F., van Heerden J.A. (1989). Nonpancreatic primary tumors with metastasis to the pancreas. Surg. Gynecol. Obstet..

[5] Kondo A., Nakmura K., Taniguchi K., Kato K., Ogura Y., Katsuta K. (2007). A case of metastatic pancreatic cancer detected and resected after the resection of cancer of the gastric remnant. J. Jpn. Surg. Assoc..

[6] Hashimoto T., Soyama K., Seshimo A., Tani H., Kambe T., Shibata N. (2007). A case of isolated metastatic pancreatic cancer from postoperative gastric cancer. J. Tokyo Women Med. Univ..

[7] Teshima S., Saito T., Yunome G., Endo A., Kikuchi S., Suzuki H. (2008). A case of gastric cancer metastasized to the pancreas treated surgically. J. Jpn. Surg. Assoc..

[8] Nakae S., Yamamoto M., Ohshima T., Okazaki T., Toyoda M., Senzaki H. (2010). A case of surgically-treated pancreatic metastasis from gastric cancer. J. Jpn. Surg. Assoc..

[9] Matsumoto M., Yasuda T., Ishikawa H., Shinkai M., Nakai T., Takeyama Y. (2012). A case of surgically resected pancreatic metastasis from gastric cancer. J. Jpn. Surg. Assoc..

[10] Watanabe S., Yamaguchi R., Itou A., Sasamoto A., Aizu K., Hayashi Y. (2014). Pancreatic metastasis from gastric cancer with portal vein tumor thrombus. Jpn. J. Gastroenterol. Surg..

[11] Crippa S., Angelini C., Mussi C., Bonardi C., Romano F., Sartori P. (2006). Surgical treatment of metastatic tumors to the pancreas: a single center experience and review of the literature. World J. Surg..

[12] Agha R.A., Borrelli M.R., Farwana R., Koshy K., Fowler A., Orgill D.P., SCARE Group (2018). The SCARE 2018 statement: updating consensus surgical CAse REport (SCARE) guidelines. Int. J. Surg..

[13] Adsay N.V., Andea A., Basturk O., Kilinc N., Nassar H., Cheng J.D. (2004). Secondary tumors of the pancreas: an analysis of a surgical and autopsy database and review of the literature. Virchows Arch..

[14] Nakamura E., Shimizu M., Itoh T., Manabe T. (2001). Secondary tumors of the pancreas: clinicopathological study of 103 autopsy cases of Japanese patients. Pathol. Int..

[15] Tsitouridis I., Diamantopoulou A., Michaelides M., Arvanity M., Papaioannou S. (2010). Pancreatic metastases: CT and MRI findings. Diagn. Interv. Radiol..

[16] Z’graggen K., Fernández-del Castillo C., Rattner D.W., Sigala H., Warshaw A.L. (1998). Metastases to the pancreas and their surgical extirpation. Arch. Surg..

